# CT-based radiomics for differentiating intracranial contrast extravasation from intraparenchymal haemorrhage after mechanical thrombectomy

**DOI:** 10.1007/s00330-022-08541-9

**Published:** 2022-02-03

**Authors:** Xiaojun Chen, Yuanzhe Li, Yongjin Zhou, Yan Yang, Jiansheng Yang, Peipei Pang, Yi Wang, Jianmin Cheng, Haibo Chen, Yifan Guo

**Affiliations:** 1 Department of Radiology, Affiliated Jinhua Hospital, Zhejiang University School of Medicine, 365 Renmin East Road, Jinhua, 321000 China; 2 CT/MRI Department, The Second Affiliated Hospital, Fujian Medical University, 34 Zhongshan North Road, Quanzhou, 362000 China; 3 Department of Radiology, Lishui Hospital of Zhejiang University, 289 Kuocang Road, Lishui, 323000 China; 4 Department of Radiology, The Second Affiliated Hospital and Yuying Children’s Hospital of Wenzhou Medical University, 109 Xueyuan West Road, Wenzhou, 325000 China; 5 Department of Neurology, School of Medicine, The Second Affiliated Hospital of Zhejiang University, 88 Jiefang Road, Hangzhou, 325000 China; 6 Department of Pharmaceuticals Diagnosis, GE Healthcare, 122 Shuguang Road, Hangzhou, 310000 China; 7 Department of Radiology, The First Affiliated Hospital of Zhejiang Chinese Medical University (Zhejiang Provincial Hospital of Traditional Chinese Medicine), 54 Youdian Road, Hangzhou, 310000 China; 8 The First School of Clinical Medicine, Zhejiang Chinese Medical University, 548 Binwen Road, Hangzhou, 310000 China

**Keywords:** Radiomics, Tomography, X-ray computed, Thrombectomy, Intracranial haemorrhage, Extravasation of diagnostic and therapeutic materials

## Abstract

**Objective:**

To develop a nonenhanced CT-based radiomic signature for the differentiation of iodinated contrast extravasation from intraparenchymal haemorrhage (IPH) following mechanical thrombectomy.

**Methods:**

Patients diagnosed with acute ischaemic stroke who underwent mechanical thrombectomy in 4 institutions from December 2017 to June 2020 were included in this retrospective study. The study population was divided into a training cohort and a validation cohort. The nonenhanced CT images taken after mechanical thrombectomy were used to extract radiomic features. The maximum relevance minimum redundancy (mRMR) algorithm was used to eliminate confounding variables. Afterwards, least absolute shrinkage and selection operator (LASSO) logistic regression was used to generate the radiomic signature. The diagnostic performance of the radiomic signature was evaluated by the area under the curve (AUC), accuracy, specificity, sensitivity, positive predictive value (PPV), and negative predictive value (NPV).

**Results:**

A total of 166 intraparenchymal areas of hyperattenuation from 101 patients were used. The areas of hyperattenuation were randomly allocated to the training and validation cohorts at a ratio of 7:3. The AUC of the radiomic signature was 0.848 (95% confidence interval (CI) 0.780–0.917) in the training cohort and 0.826 (95% CI 0.705–0.948) in the validation cohort. The accuracy of the radiomic signature was 77.6%, with a sensitivity of 76.7%, a specificity of 78.9%, a PPV of 85.2%, and a NPV of 68.2% in the validation cohort.

**Conclusions:**

The radiomic signature constructed based on initial post-operative nonenhanced CT after mechanical thrombectomy can effectively differentiate IPH from iodinated contrast extravasation.

**Key Points:**

• *Radiomic features were extracted from intraparenchymal areas of hyperattenuation on initial post-operative CT scans after mechanical thrombectomy.*

• *The nonenhanced CT-based radiomic signature can differentiate IPH from iodinated contrast extravasation early.*

• *The radiomic signature may help prevent unnecessary rescanning after mechanical thrombectomy, especially in cases where contrast extravasation is highly suggestive.*

## Introduction

Mechanical thrombectomy has a high recanalisation rate for large vessel occlusion, significantly improves the functional independence of patients [1–7], and may provide the opportunity for recanalisation of patients who do not meet the strict eligibility criteria for intravenous thrombolysis [8]. However, the rate of symptomatic intracerebral haemorrhage after mechanical thrombectomy ranges from 0 to 7.7% [9]. Early haemorrhagic transformation after treatment may continue to evolve, leading to a marked deterioration in some patients [10–12]. Therefore, the early identification of intraparenchymal haemorrhage (IPH) after mechanical thrombectomy provides the possibility to limit haemorrhagic growth, which can provide significant value in the management of post-operative patients [12, 13].

Following mechanical thrombectomy, nonenhanced computed tomography (CT) scans are often performed to determine the success of therapy, especially the presence of intracerebral haemorrhage. In this case, intraparenchymal hyperattenuation on CT after mechanical thrombectomy is a common occurrence. However, it is difficult to distinguish intracerebral haemorrhage from contrast extravasation on CT because of the similar radiologic appearance of both pathologies. The contrast remains visible on imaging for 24 + h before it is cleared from circulation [14]. Therefore, the persistence of hyperattenuation on CT after 24 h can be considered haemorrhage [15]. However, patients presenting with hyperattenuation between 0 and 24 h after mechanical thrombectomy fit within a diagnostic grey area. As such, life-saving treatments such as anticoagulation or antiplatelet therapy are often delayed [16, 17].

Radiomics is a powerful method to extract key parameters from medical images and is a powerful tool for guiding diagnosis and treatment in modern medicine [18–20]. Currently, the application of radiomics in intracerebral haemorrhage is a growing field, and quantitative analysis of haematoma heterogeneity using radiomics is a feasible method [21–24]. As contrast material and blood contents have different viscosities, radiomics may be used to determine the composition of hyperattenuation on CT following mechanical thrombectomy.

This study was designed to determine the possibility of using radiomics to delineate IPH (haemorrhage alone and mixed haemorrhage and iodinated contrast material) from iodinated contrast extravasation.

## Materials and methods

### Patient selection

This retrospective study was approved by our institutional review board, which waived the requirement for informed consent. From December 2017 to June 2020, 257 patients with acute ischaemic stroke who underwent mechanical thrombectomy at four institutions were retrospectively enrolled, including 30 patients from The First Affiliated Hospital of Zhejiang Chinese Medical University, 55 patients from the Affiliated Jinhua Hospital, Zhejiang University School of Medicine, 114 patients from The Second Affiliated Hospital and Yuying Children’s Hospital of Wenzhou Medical University, and 58 patients from the Lishui Hospital of Zhejiang University. The inclusion criteria were as follows: (1) patients underwent nonenhanced head CT after mechanical thrombectomy; (2) initial post-operative nonenhanced head CT was performed within 1 h after mechanical thrombectomy; and (3) intraparenchymal area of hyperattenuation, which was defined as an area with an objectively higher density than the surrounding grey or white matter [25], could be seen on the initial nonenhanced head CT after mechanical thrombectomy. The exclusion criteria were as follows: (1) the follow-up time of nonenhanced head CT after mechanical thrombectomy was less than 24 h; (2) artefacts (e.g. metal artefacts or motion artefacts) [26] affected the intraparenchymal areas of hyperattenuation in CT images; and (3) patients underwent craniotomy after mechanical thrombectomy, which made it difficult to identify the intraparenchymal area of hyperattenuation.

All intraparenchymal areas of hyperattenuation of the included patients were randomly divided into training and validation cohorts (7:3). Stratified random sampling was used to keep the same proportion of IPH and iodinated contrast extravasation in both the training and validation cohorts.

### Image interpretation

The intraparenchymal area of hyperattenuation was considered to represent iodinated contrast extravasation if the area of hyperattenuation was entirely or almost entirely cleared within 24–48 h after the procedure. If the area of hyperattenuation persisted or increased for more than 48 h, it was classified as IPH [25, 27]. The intraparenchymal area of hyperattenuation may remain after 24–48 h in patients with renal dysfunction [28]. According to the pharmacokinetics of iodinated contrast agents and CT imaging of intracranial haemorrhage [28–30], the hyperattenuating area of patients with renal dysfunction was considered to represent contrast extravasation if it was entirely or almost entirely cleared within 7 days after the procedure. All nonenhanced CT images were analysed by two experienced radiologists.

### CT data acquisition

All images of the initial post-operative nonenhanced head CT were obtained from 7 multislice CT scanners (Aquilion ONE, Toshiba Medical Systems; Brilliance 16 or Brilliance iCT, Philips Healthcare; SOMATOM Definition AS + , SOMATOM Emotion 16 or SOMATOM Force, Siemens Healthcare; uCT 710, United Imaging Healthcare). The scanning parameters were as follows: tube voltage: SOMATOM Force 90 kVp, SOMATOM Emotion 16 130 kVp, other CT scanners 120 kVp; tube current: SOMATOM Definition AS + 226 mA, uCT 710 228 mA, Aquilion ONE 230 mA, Brilliance 16 250 mA, Brilliance iCT 400 mA, other CT scanners automatic tube current modulation; matrix size: 512 × 512; field of view (FOV): Brilliance 16 250 mm, other CT scanners FOV was adapted to the patient size; section thickness: Brilliance 16 and SOMATOM Definition AS + 6 mm, SOMATOM Emotion 16 9.6 mm, other CT scanners 5 mm; section interval: Brilliance 16 and SOMATOM Definition AS + 6 mm, SOMATOM Emotion 16 9.6 mm, other CT scanners 5 mm.

### ROI segmentation

**Fig. 1:**
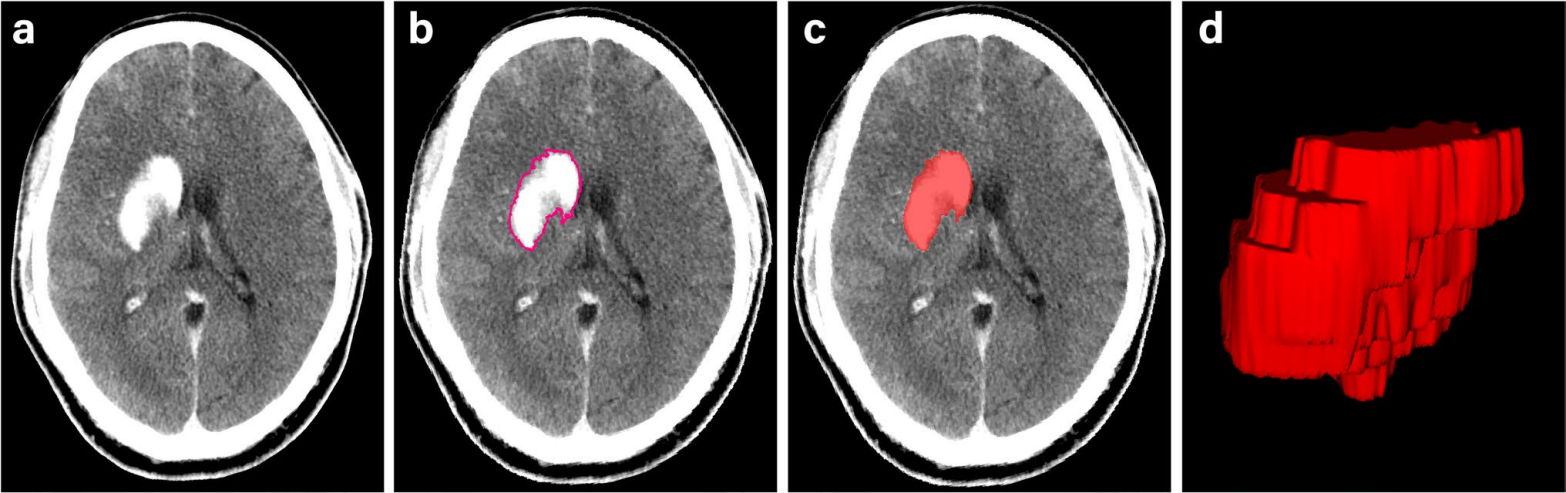
Delineation of the hyperattenuating areas using ITK-SNAP software. **a** A hyperattenuating area in the left lentiform nucleus on nonenhanced CT. **b** Manual segmentation along the hyperattenuating area contour on the transverse section. **c** The ROI of the hyperattenuating area on a transverse section is displayed as the red area. **d** 3D ROI for the whole hyperattenuating area. 3D three-dimensional, CT computed tomography, ROI region of interest

All regions of interest (ROIs) were determined using ITK-SNAP 3.6.0 [31]. One radiologist (reader A, with 6 years of experience in diagnostic neuroradiology and blinded to the ultimate diagnosis) manually segmented the images along the intraparenchymal areas of the hyperattenuation contour on each transverse section (Fig. 1). Referring to a guideline of selecting and reporting intraclass correlation coefficients (ICCs) for reliability research [32], thirty lesions were randomly selected to evaluate the intraobserver and interobserver agreement of feature extraction. The 30 lesions were re-segmented by reader A 1 month later to evaluate the intraobserver agreement. A senior radiologist (reader B, with more than 10 years of experience in diagnostic neuroradiology, also blinded to the ultimate diagnosis) re-segmented the 30 lesions to evaluate the interobserver agreement. Intraclass and interclass ICCs were used to determine the intraobserver and interobserver agreement of feature extraction. Any ICCs larger than 0.80 were considered to indicate good agreement.

### Radiomic feature extraction

A total of 1316 radiomic features were extracted using the PyRadiomics module inserted in AK software (Artificial Intelligence Kit; GE Healthcare) [33]. All extracted features can be subdivided into the following classes: first order statistics, shape-based, grey level co-occurrence matrix (GLCM), grey level size zone matrix (GLSZM), grey level run length matrix (GLRLM), neighbouring grey tone difference matrix (NGTDM), and grey level dependence matrix (GLDM). A detailed description of the radiomic features can be found on the PyRadiomics documentation website (http://pyradiomics.readthedocs.io).

### Radiomic model engineering

The features obtained were normalized, and the unit limit was removed. For radiomic features with high reproducibility (intraobserver and interobserver ICCs > 0.80), the maximum relevance minimum redundancy (mRMR) algorithm was used to assist in the removal of confounding factors. The extracted features were indexed in accordance with their relevance-redundancy indexes. Afterwards, the top ten features were retained. Least absolute shrinkage and selection operator (LASSO) logistic regression [34] was then applied to determine the best features to build the radiomic signature. Features with nonzero coefficients were chosen using tenfold cross-validation. The radiomics score (Rad-score) was generated by calculating the weighted sum of the features to determine the probability of haemorrhage. The radiomic signature was constructed based on the training cohort and evaluated in the validation cohort.

### Statistical analysis

Statistical analyses were performed using SPSS 24.0 software and R software (version 3.5.0; www.R-project.org). The receiver operating characteristic (ROC) curve was used to determine the diagnostic performance of both cohorts. The area under the curve (AUC), accuracy, sensitivity, specificity, positive predictive value (PPV), and negative predictive value (NPV) were then determined using the Youden index [35]. Calibration curves along with the Hosmer–Lemeshow test were used to determine the calibration of the radiomic signature [36].

## Results

### Demographics

**Fig. 2:**
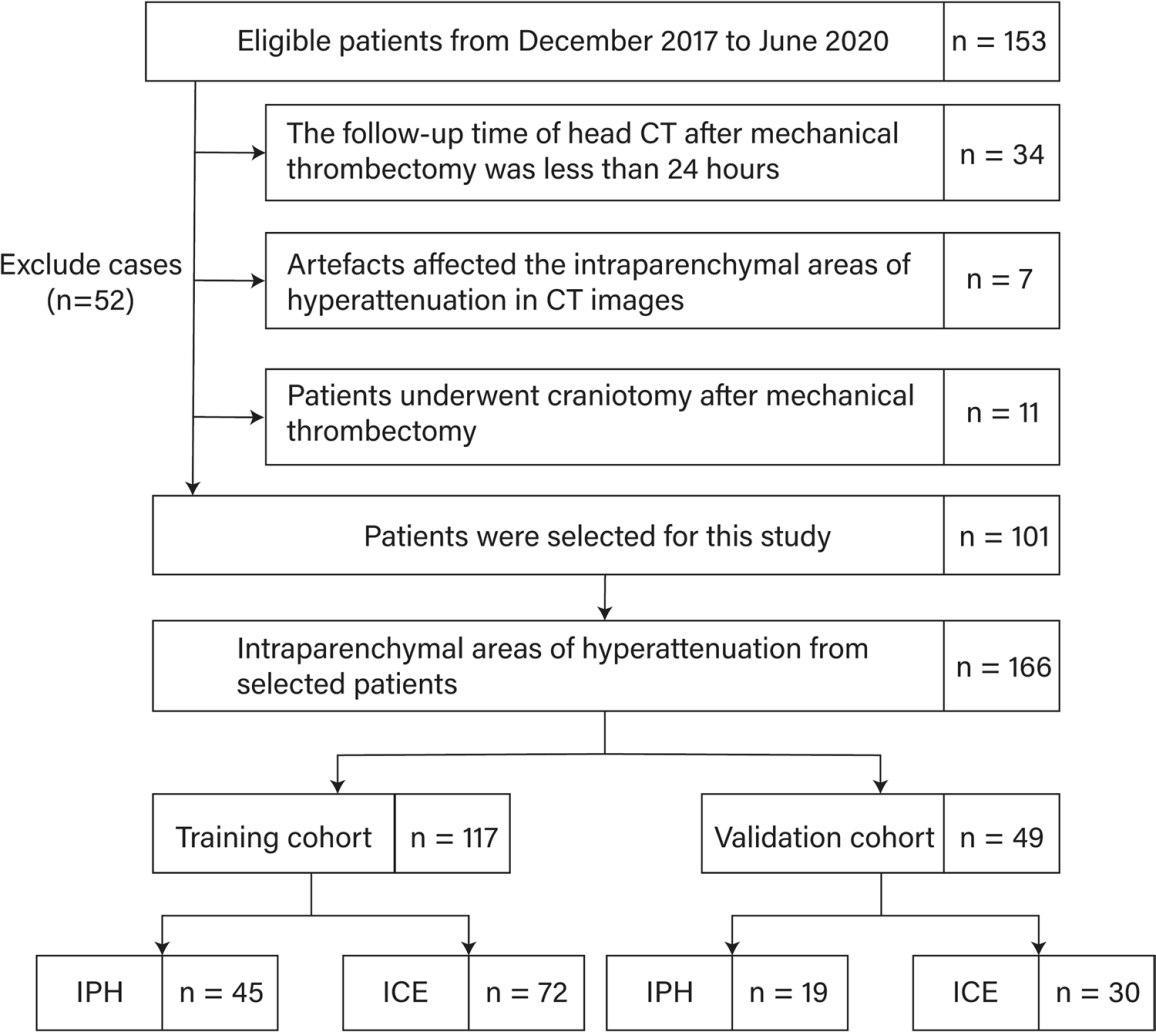
Flowchart of the study shows the recruitment pathway for patients. ICE iodinated contrast extravasation, IPH intraparenchymal haemorrhage

A total of 166 intraparenchymal areas of hyperattenuation from 101 patients were selected for this study (Fig. 2). All the areas of hyperattenuation were randomly assigned to the training cohort (*n* = 117) and the validation cohort (*n* = 49). Of the 166 intraparenchymal areas of hyperattenuation, 64 were IPH and 102 were iodinated contrast extravasation. Of the 101 patients, 64 were males and 37 were females. The mean age of the patients was 72.0 ± 11.0 years (range 39–92 years).

### Radiomic signature construction

Of the 1316 radiomic features, 981 features with intraobserver and interobserver ICCs > 0.80 were retained. Of the 981 stable radiomic features, redundant and irrelevant features were eliminated by mRMR, and 10 features were retained. The mean intraobserver and interobserver ICCs of the retained features were 0.950 (95% confidence interval (CI) 0.907–0.994) and 0.974 (95% CI 0.938–1.000), respectively. On the basis of the training cohort, the 9 most significant features were selected to build the radiomic signature by LASSO logistic regression. The equation was as follows:

$$\mathrm{Rad}-\mathrm{score }= -0.487 (\mathrm{constant}) +\mathrm{ coefficients}\times \mathrm{features}$$

**Fig. 3:**
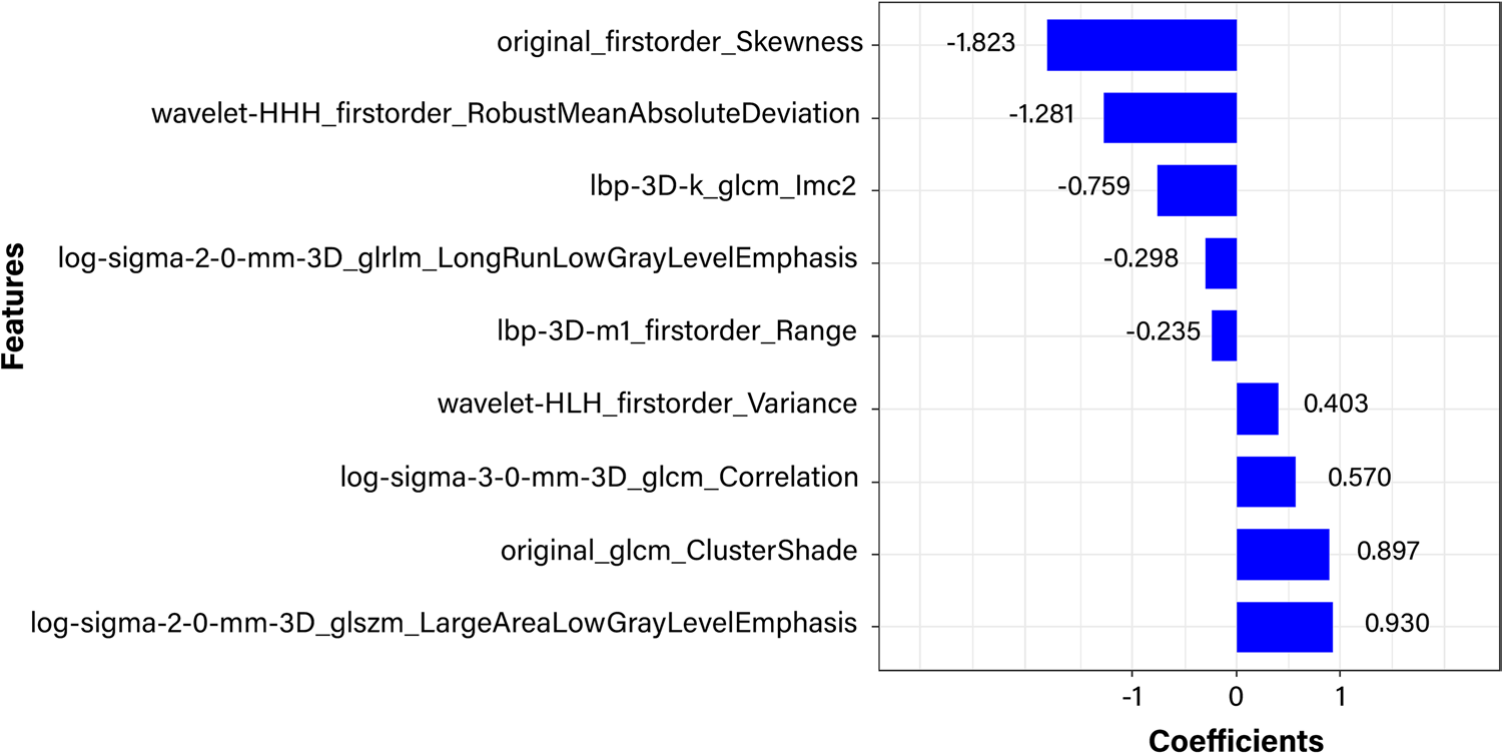
Selected radiomic features and their corresponding coefficients

**Fig. 4:**
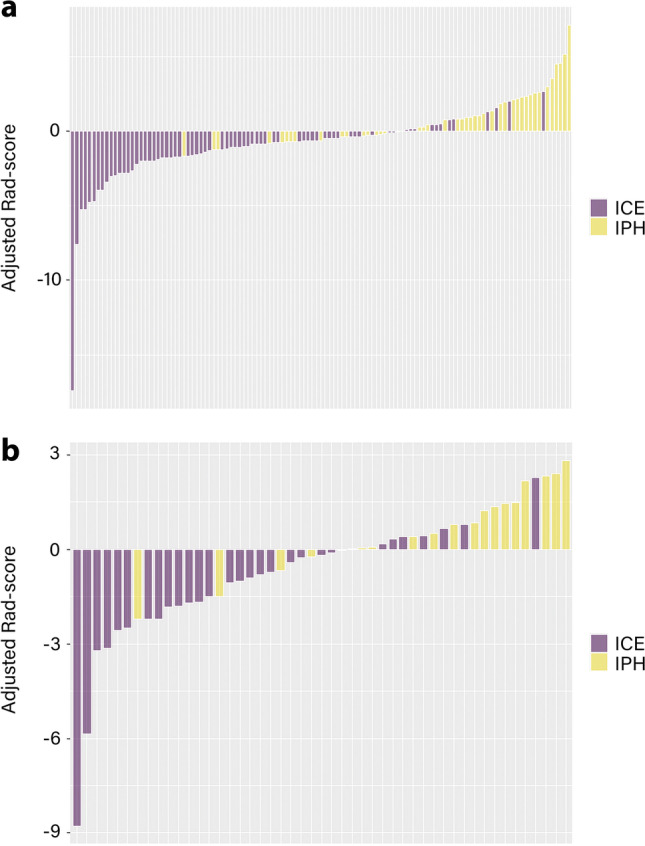
Waterfall plots of the radiomic signature. The waterfall plot shows the distribution of the adjusted Rad-scores and diagnoses of each patient in the training cohort (**a**) and the validation cohort (**b**). The default is set to IPH (the yellow bar) above the baseline and ICE (the purple bar) below the baseline. This plot depicts the association between the predicted and actual diagnoses in which mismatching of the colour coding indicates misclassification by the Rad-score. Rad-score minus the cutoff value is the adjusted Rad-score. ICE iodinated contrast extravasation, IPH intraparenchymal haemorrhage, Rad-score radiomics score

The feature names and corresponding coefficients are displayed in Fig. 3. Figure 4 shows the waterfall plots of the radiomic signature to differentiate IPH from iodinated contrast extravasation. The cutoff value of radiomic signature was − 0.365.

### Diagnostic ability of the radiomic signature

**Fig. 5:**
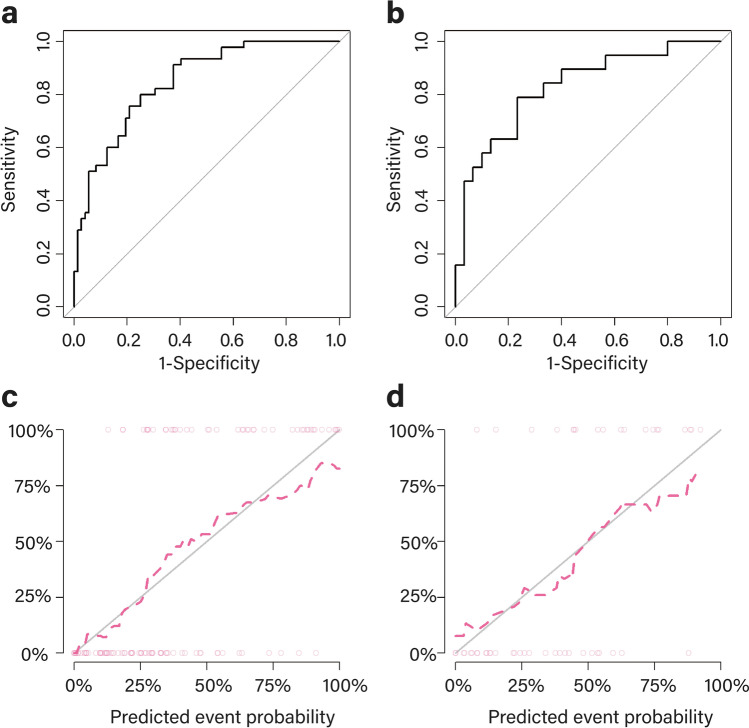
ROC curves and calibration curves of the radiomic signature in the training cohort (**a**, **c**) and the validation cohort (**b**, **d**). The 45° solid grey line of the calibration curve represents a perfect prediction, and the pink dotted line represents the predictive performance of the radiomic signature. ROC receiver operating characteristic

In the training cohort, the AUC of the radiomic signature was 0.848 (95% CI 0.780–0.917, *p* < 0.001). In the validation cohort, the AUC of the radiomic signature was 0.826 (95% CI 0.705–0.948, *p* < 0.001). Further information can be found in Table 1 and Fig. 5a and b. The calibration curve indicated a strong level of agreement between the actual and predicted diagnoses in both cohorts (Fig. 5c, d). The results of the Hosmer–Lemeshow test were not statistically significant, with a *p* value of 0.692 for the training cohort and 0.633 for the validation cohort.

**Table 1:** Diagnostic performance of the radiomic signature in the training and validation cohorts

	AUC	95% CI	Accuracy (%)	Sensitivity (%)	Specificity (%)	PPV (%)	NPV (%)
Training cohort	0.848	0.780–0.917	76.9%	75.0%	80.0%	85.7%	66.7%
Validation cohort	0.826	0.705–0.948	77.6%	76.7%	78.9%	85.2%	68.2%

*AUC* area under the curve, *CI* confidence interval, *NPV* negative predictive value, *PPV* positive predictive value

## Discussion

Intracerebral haemorrhage is a common complication following mechanical thrombectomy. IPH with a mass effect can cause clinical deterioration and greatly impair prognosis [37, 38]. The identification of IPH after mechanical thrombectomy is of great significance for the adjustment of treatment [12, 13]. In the early phase after mechanical thrombectomy, conventional CT has a limited ability to differentiate IPH from iodinated contrast extravasation [11, 39–41]. From a pathophysiological standpoint, contrast extravasation is caused by a breakdown of the blood–brain barrier [42]. The early differentiation between IPH and iodinated contrast extravasation is important for the adjustment of therapy after mechanical thrombectomy, which will determine whether anticoagulation or antiplatelet therapy should be performed as early as possible [16, 17]. Therefore, this study attempted to use radiomics to help rapidly diagnose the composition of hyperattenuation to guide further treatment.

In this study, a preliminarily constructed radiomic signature based on initial post-operative nonenhanced CT after mechanical thrombectomy to differentiate IPH from iodinated contrast extravasation was used. ROC analysis revealed that the performance of the radiomic model was high. In the training and validation cohorts, the AUCs of this radiomic signature were 0.848 and 0.826, respectively. The results indicated that there was a significant difference in heterogeneity between IPH and iodinated contrast extravasation. The selected radiomic features may be able to predict the differences between IPH and contrast extravasation on nonenhanced CT images.

Conventional CT imaging is a cornerstone of diagnostic imaging due to its ease of access. Xu et al. determined that noncontrast CT performed immediately following mechanical thrombectomy can predict parenchymal haemorrhage at 24 h with metallic hyperdensity, but this sign was only visible in some patients [41]. It is still difficult to differentiate IPH from iodinated contrast extravasation based on conventional CT. Our radiomic signature was constructed based on routine CT scans and can be used to diagnose all intraparenchymal areas of hyperattenuation, proving its versatility. Magnetic resonance imaging (MRI) is costly in both time and financial capital in terms of obtaining interpretable images [17]. However, dual-energy CT is able to reliably diagnose intracerebral haemorrhage vs. contrast extravasation [25, 27, 43]. This radiomic signature may serve as a practical solution for clinics without dual-energy CT scanners or situations where dual-energy CT examinations were not performed. The predictive result of the radiomic signature can be obtained based on the initial post-operative nonenhanced CT to prevent unnecessary rescanning, especially in cases where contrast extravasation is highly suggestive. Therefore, the use of radiomics on nonenhanced CT images to differentiate IPH from iodinated contrast extravasation can improve both the accuracy of and access to diagnostic tools.

The CT images used to construct the radiomic signature were obtained from 7 multislice CT scanners at 4 institutions, resulting in a variety of CT acquisition parameters. The use of multivendor images to evaluate artificial intelligence algorithms is advocated [44]. However, differences between scanners and between image acquisition settings limit the predictive potential of radiomic models [45–47]. The predictive ability of radiomic models may be improved by using stringent image acquisition protocols or correcting for the parameters of the scanner during data analysis [45, 47]. Therefore, further optimisation of CT acquisition parameters may improve the performance of radiomic signature–based diagnostic systems.

While the findings presented herein offer promising insight, several limitations are present. As a small study, the sample size is one issue. As this study was retrospective, follow-up CT data after mechanical thrombectomy were often insufficient. Therefore, further external validation should be performed in a prospective study with a larger cohort. Second, all ROIs in this study were manually segmented, which is an intensive process. Automatic segmentation still has a long way to go before its accuracy and reproducibility rival those of manual segmentation. Finally, the diagnostic performance of our algorithm in distinguishing contrast extravasation from IPH was not validated. The diagnostic performance of radiomic models constructed by other algorithms needs to be further studied.

In conclusion, our nonenhanced CT-based radiomic signature can effectively differentiate IPH from iodinated contrast extravasation in the early phase after mechanical thrombectomy, which may be helpful for the post-operative management of patients.

## Abbreviations

AUC: Area under the curve
CI: Confidence interval
CT: Computed tomography
FOV: Field of view
GLCM: Grey level co-occurrence matrix
GLDM: Grey level dependence matrix
GLRLM: Grey level run length matrix
GLSZM: Grey level size zone matrix
ICC: Interclass correlation coefficient
IPH: Intraparenchymal haemorrhage
LASSO: Least absolute shrinkage and selection operator
MRI: Magnetic resonance imaging
mRMR: Maximum relevance minimum redundancy
NPV: Negative predictive value
NGTDM: Neighbouring grey tone difference matrix
PPV: Positive predictive value
ROC: Receiver operating characteristic
ROI: Region of interest

## Declarations

### Guarantor

The scientific guarantor of this publication is Yifan Guo.

### Statistics and biometry

One of the authors (Peipei Pang) has significant statistical expertise.

### Methodology

• retrospective

• diagnostic or prognostic study

• multicentre study
